# Heterostructured Electrocatalysts for Hydrogen Evolution Reaction Under Alkaline Conditions

**DOI:** 10.1007/s40820-018-0229-x

**Published:** 2018-11-07

**Authors:** Jumeng Wei, Min Zhou, Anchun Long, Yanming Xue, Hanbin Liao, Chao Wei, Zhichuan J. Xu

**Affiliations:** 1 College of Chemistry and Materials Engineering, Anhui Science and Technology University, Bengbu, 233100 People’s Republic of China; 2 College of Physical Science and Technology, and Institute of Optoelectronic Technology, Yangzhou University, Yangzhou, 225002 People’s Republic of China; 3 School of Materials Science and Engineering, Hebei University of Technology, Tianjin, 300130 People’s Republic of China; 4 School of Materials Science and Engineering, Nanyang Technological University, 50 Nanyang Avenue, Singapore, 639798 Singapore; 5 The Cambridge Centre for Advanced Research and Education in Singapore, 1 CREATE Way, Singapore, 138602 Singapore; 6 Solar Fuels Laboratory, Nanyang Technological University, 50 Nanyang Avenue, Singapore, 639798 Singapore; 7 Energy Research Institute @ Nanyang Technological University, 50 Nanyang Avenue, Singapore, 639798 Singapore

**Keywords:** Hybrid catalyst, Hydrogen production, Water splitting, Interface engineering, Synergistic effect

## Abstract

The hydrogen evolution reaction (HER) is a half-cell reaction in water electrolysis for producing hydrogen gas. In industrial water electrolysis, the HER is often conducted in alkaline media to achieve higher stability of the electrode materials. However, the kinetics of the HER in alkaline medium is slow relative to that in acid because of the low concentration of protons in the former. Under the latter conditions, the entire HER process will require additional effort to obtain protons by water dissociation near or on the catalyst surface. Heterostructured catalysts, with fascinating synergistic effects derived from their heterogeneous interfaces, can provide multiple functional sites for the overall reaction process. At present, the activity of the most active known heterostructured catalysts surpasses (platinum-based heterostructures) or approaches (noble-metal-free heterostructures) that of the commercial Pt/C catalyst under alkaline conditions, demonstrating an infusive potential to break through the bottlenecks. This review summarizes the most representative and recent heterostructured HER catalysts for alkaline medium. The basics and principles of the HER under alkaline conditions are first introduced, followed by a discussion of the latest advances in heterostructured catalysts with/without noble-metal-based heterostructures. Special focus is placed on approaches for enhancing the reaction rate by accelerating the Volmer step. This review aims to provide an overview of the current developments in alkaline HER catalysts, as well as the design principles for the future development of heterostructured nano- or micro-sized electrocatalysts.
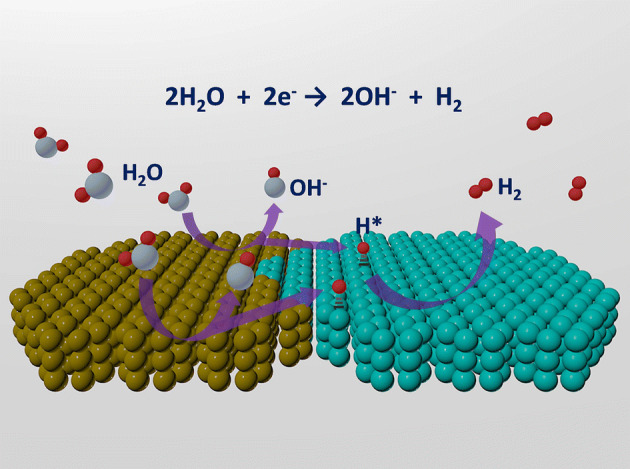

## Highlights

- Heterostructured catalysts offer improved HER activities in alkaline solutions due to their fascinating synergistic effects on the heterogeneous interfaces.
- Representative heterostructured HER catalysts for alkaline media are summarized.
- Acceleration of the Volmer step, crucial for alkali-active catalysts, is highlighted.

## Introduction

The ever-increasing demands for energy have led to the massive consumption of fossil fuels worldwide, with accompanying concerns about their limited reserves and serious environmental issues. During the past decades, alternative energy resources, including solar, wind, and hydroelectric power, have been considered as clean and sustainable alternatives to fossil fuels. However, the utilization of these clean energy resources is restricted by their uneven spatial and temporal distribution; hence, highly efficient technologies for energy storage and conversion are desired. Hydrogen, an eco-friendly fuel with high energy density, is recognized as a promising medium for storage of the energy from these sustainable resources [1, 2]. Thus, the acquisition of hydrogen via a low-cost and highly efficient route is a significant issue and has long been studied.

To date, the main route for industrial hydrogen production is the electrolysis of water in alkaline solution, which involves the hydrogen evolution reaction (HER) on the cathode and the oxygen evolution reaction (OER) on the anode within an electrolyzer [3, 4]. The water splitting efficiency depends on the catalytic activity of the electrocatalysts utilized on both electrodes [5–8]. Specifically, the HER in alkaline solution suffers from relatively slow kinetics compared to that in acidic solution, thus requiring a high overpotential to drive the reaction [9–12]. Further, the alkaline HER is also a key step in the chlor-alkali process, a widely applied but energy-intensive practice. Therefore, the design and fabrication of high-performance HER catalysts for alkaline medium are undertakings of great significance for reducing energy consumption.

The sluggish reaction rate of the HER in alkaline solution originates from the additional water dissociation step that provides protons for the subsequent reactions, but this step does not occur in acidic solution. Generally, in alkaline medium, the HER proceeds through two steps: First, the catalyst cleaves a H_2_O molecule into a hydroxyl ion (OH^−^) and an adsorbed hydrogen atom (H_ads_, Volmer step), and then a hydrogen molecule detaches via either the interaction of the H atom and water molecule (Heyrovsky step), or the combination of two H atoms (Tafel step). The catalyst should facilitate the reactions in both steps, each of which involves diverse adsorbed intermediates, making optimization of the catalytic activity difficult [11]. Even for the most prominent catalyst, Pt, the catalytic activity in alkaline medium is hindered by the sluggish water dissociation step, resulting in a reaction rate that is 2–3 orders of magnitude lower than that in acidic solution [11, 13, 14]. Though much effort has been expended in this research area, there is an emerging desire from academics to industry to enhance the catalytic performance of the state-of-the-art catalysts for the alkaline HER.

Recently, heterostructured materials, especially those on the nanoscale, have exhibited great potential in this area and drawn particular attention from researchers. These classes of catalysts, with double or multiple types of active sites on the surface, exhibit remarkable advantages for the HER in alkaline solutions. For instance, in Pt/Ni(OH)_2_ heterostructured catalysts, Ni(OH)_2_ provides active sites for cleaving the H–OH bonds, while Pt facilitates combination of the generated hydrogen intermediates to form H_2_ molecules [15]. This type of bicomponent material displays significantly enhanced catalytic activity compared to those of the corresponding unitary components. In addition, each building block in these heterostructured catalysts is adjustable, allowing for the study of a large number of combinations. Moreover, an electronic effect between the two components at the interface of the heterostructures has been revealed, which may play a crucial role in modifying the electronic structure of the monomers and thus provide more possibilities for tuning the catalytic performance of the alkaline HER.

Although many reported heterostructured catalysts have demonstrated enhanced catalytic activity, there still are many drawbacks from the heterostructural combination. For example, the heterostructured catalysts still display limited catalytic performance as their active sites are merely localized on the limited interfaces of the two components [14, 16]. Besides, though improvement on either part of the heterostructure leads to a promotion of the catalytic activity, identification of the key component that relates to the rate-determining step has not been achieved, and no guidelines for designing such catalysts have been established. Moreover, because of the high price and limited reserves of Pt, some non-noble-metal-based heterostructures have been explored as low-cost HER catalysts [17]. However, there is an obvious gap between the catalytic performance of Pt-based and noble-metal-free catalysts. Therefore, to address these issues, several strategies for fabricating various types of heterostructured catalysts on the nanoscale have been adopted. Thus, summarizing the recent advances in the design and fabrication of advanced heterostructured catalysts is of significance for developing better catalysts.

In this review, the basics and principles of the HER under alkaline conditions are first introduced. Heterostructured catalysts for alkaline HER are then discussed in terms of noble-metal-based heterostructured catalysts and non-noble-metal-based heterostructured catalysts. In addition, several emerging strategies for expediting the Volmer step in alkaline media are summarized. This review article aims to provide an overview of the mechanisms of activity promotion for heterostructured catalysts and the corresponding syntheses. The popular design principles summarized herein may help readers to further develop their own multifunctional electrocatalysts, as well as new nanosized catalyst design strategies, for the alkaline HER.

## Basics and Principles for HER Under Alkaline Conditions

The HER is a two-electron transfer reaction, in which active catalysts are required to reduce the energy barriers in each step. The generally accepted pathways for the HER in acidic solution are associated with the adsorption/desorption of a hydrogen intermediate (H*) through either the Volmer–Heyrovsky or the Volmer–Tafel mechanism [11–13]:

1 $${\text{Volmer}}\;{\text{step:}}\;{\text{H}}^{ + } + {\text{e}}^{-} \to {\text{ H}}^{*}$$

2 $${\text{Heyrovsky}}\;{\text{step: H}}^{*} + {\text{H}}^{ + } + {\text{e}}^{-} \to {\text{ H}}_{2}$$

3 $${\text{Tafel}}\;{\text{ step: H}}^{*} + {\text{ H}}^{*} \, \to {\text{ H}}_{2}$$

In alkaline medium, the reaction formula for the HER is represented as:  

4 $${\text{Volmer}}\;{\text{ step: H}}_{2} {\text{O }} + {\text{ e}}^{-} \to {\text{ H}}^{*} \, + {\text{ OH}}^{ - }$$

5 $${\text{Heyrovsky}}\;{\text{ step: H}}_{2} {\text{O }} + {\text{ e}}^{-} + {\text{ H}}^{*} \, \to {\text{ H}}_{2} + {\text{OH}}^{ - }$$

6 $${\text{Tafel }}\;{\text{step: H}}^{*} + {\text{ H}}^{*} \, \to {\text{ H}}_{2}$$

**Scheme 1:**
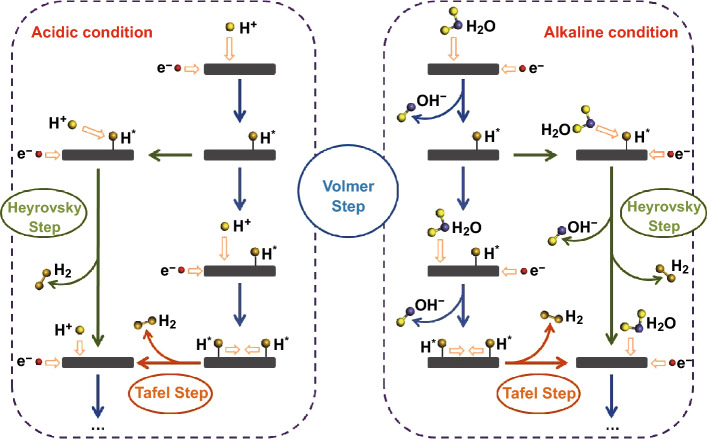
Schematic pathways for hydrogen evolution reaction under acidic and alkaline conditions

The pathways for the HER under acidic and alkaline conditions are illustrated in Scheme 1. In general, the energy required to drive the overall reaction differs based on the reaction pathways involved, where the pathways have different energy barriers. In fact, catalysts display obviously lower activity and exchange current densities in alkaline solution than in acidic solution [18, 19]. For example, the experimentally obtained reaction rate for the HER with Pt in alkaline medium is two or three orders of magnitude lower than that under acidic conditions. This is likely caused by the initial water dissociation process in the Volmer step of the alkaline HER, which supplies H* to the following steps by cleaving the H–O–H bond, and is considered to be the rate-determining step of the overall reaction.

Although the catalytic activity of HER catalysts strongly depends on the pH of the electrolyte, the underlying mechanisms are still under debate [20]. Conway et al. [21, 22] investigated the effects of underpotential- (UPD) and overpotential-deposited (OPD) H on the Pt surface based on the ‘volcano relation’ between the exchange current density and the bond energy of H chemisorbed on the metal or the standard Gibbs energies of chemisorption of 2H from H_2_. They found that the kinetically significant intermediate adsorbed on Pt and related metals in the HER is the weakly bound OPD H rather than the strongly bound UPD-type of H. The more the OPD H, the higher the activity. The OPD H coverage is much lower in alkaline than acidic solutions, making the discharge step (Volmer step) the rate-controlling step in alkaline solution, which results in a slower reaction rate. Lately, Yan et al. [23] have proposed that the activity of a catalyst for the HER relies solely on the H-binding energy (HBE) of the electrocatalysts, which is sensitive to the pH of the electrolyte. In an electrolyte with a higher pH, the HBE of the catalyst is larger, thus resulting in slower reaction kinetics of the HER in base. Nevertheless, further understanding of how the pH affects the HBE has not been achieved. Moreover, this model is inadequate for interpreting the catalytic behavior on well-defined single-crystal surfaces, such as Pt (111) surfaces [24]. In contrast, Koper and Markovic et al. insisted that the catalytic activity is not solely associated with the HBE; other factors, such as adsorption of the hydroxyl (OH*) intermediate, need to be taken into account [25–27]. Either way, a detailed explanation on the atomic and molecular level is desired to address this issue.

Despite the controversies mentioned above, practical strategies have been developed to promote the HER performance in alkaline solutions. Alloying of transition metals has been found to be an effective route for enhancing the activity of catalysts for the alkaline HER. In the 1980s, Brown and Aruraj et al. investigated the activity of nickel-based binary and ternary alloy catalysts for the HER, where the nickel–molybdenum alloy outperformed the congeners in alkaline medium [28, 29]. Most recently, benefitting from the optimization of the microstructure and composites, Ni_4_Mo nanoparticles exhibited excellent catalytic activity, comparable to that of the commercial Pt/C catalyst [30–32]. However, due to the dissolution of metallic molybdenum under the intermittent electrolyzing conditions in alkaline solution, the practical application of such alloys is limited by their poor stability. On the other hand, heterostructured nanomaterials have drawn much attention in this area. Markovic’s group first deposited nickel hydroxide nanoclusters on a Pt electrode and demonstrated a sevenfold enhancement of the HER catalytic activity in 1 M KOH solution [15]. Since then, many attempts have been made to fabricate heterostructured catalysts, aiming to construct ideal candidates that minimize or circumvent the use of noble metals and exhibit improved HER catalytic performance, as discussed hereinafter.

## Heterostructured Electrocatalysts for Alkaline HER

### Noble-Metal-Based Heterostructured Catalysts

**Fig. 1:**
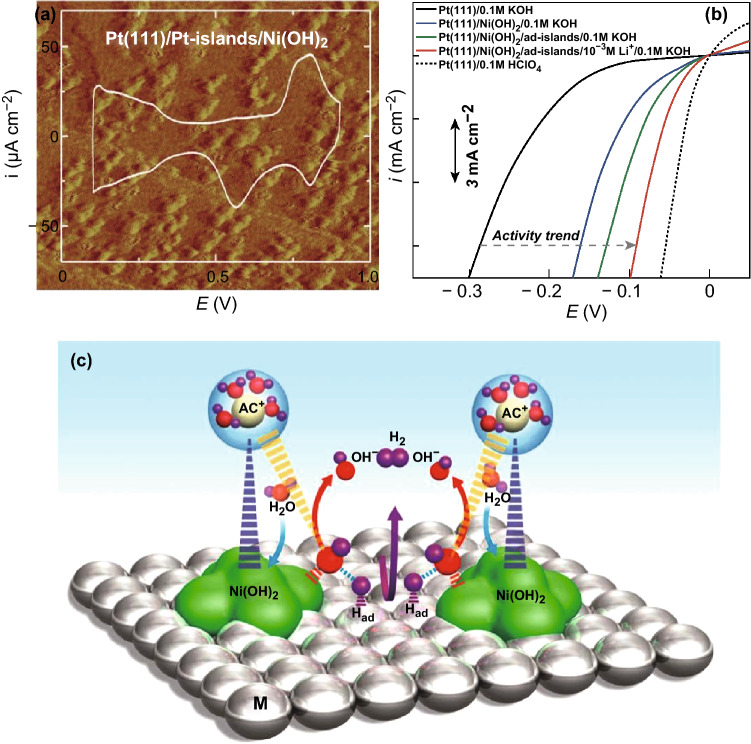
**a** STM image (60 nm × 60 nm) and CV trace of the Pt/Ni(OH)_2_ surface. **b** Comparison of HER activities of Ni(OH)_2_-modified Pt electrode and control samples in 0.1 M KOH. **c** Schematic representation of water dissociation, formation of M–H_ad_ intermediates, and subsequent recombination of two H_ad_ atoms to form H_2_ (magenta arrow), as well as OH^−^ desorption from the Ni(OH)_2_ domains (red arrows) followed by adsorption of another water molecule on the same site (blue arrows). Reproduced with permission of the authors of Ref. [15]. Copyright 2011, American Association for the Advancement of Science. (Color figure online)

As discussed, the alkaline HER involves two steps, i.e., the water dissociation step to produce adsorbed H*, and the generation of H_2_ via either the Heyrovsky or the Tafel step. The commercial Pt catalyst has been found ineffective for accelerating the water dissociation step, though it is a well-documented active material for the latter. In 2011, Markovic et al. designed a composite material by depositing ultrathin Ni(OH)_2_ clusters on a Pt electrode, wherein Ni(OH)_2_ provides the active sites for water dissociation and Pt facilitates the adsorption of atomic hydrogen and subsequent desorption of the formed H_2_ [15]. Benefitting from the synergistic effect between the two components, this heterostructured catalyst displayed enhanced catalytic activity (by a factor of ~ 7) for the HER in KOH solution, compared to the pristine Pt surface catalyst (Fig. 1). Moreover, the performance of the Pt/Ni(OH)_2_ composite was further improved by introducing solvated Li^+^ ions into the compact portion of the double layer, promoting the efficiency of the water dissociation step.

Because the HER takes place on the interfaces of the heterostructures, enlarging the interface density is a reasonable route for providing more active sites and improving the catalytic performance. Huang’s group prepared a NiO_*x*_/Pt–Ni heterostructure by annealing metallic Pt–Ni nanowires in air, during which the segregated Ni in the Pt–Ni alloy was oxidized into NiO_*x*_ shells that aggregated on the exterior surface of the nanowires [33]. The density of NiO_*x*_ could be controlled by tuning the Ni content of the precursors and should be neither too low, nor too high in order to maximize the interface between the two components. By using Pt_3_Ni nanowires as a precursor, NiO_*x*_/Pt_3_Ni with a proper NiO_*x*_ density was obtained and furnished higher HER activity than commercial Pt/C in 0.1 M and 1 M KOH. Subsequently, they constructed a Pt_3_Ni_2_/NiS interface nanowire by sulfurization of the composition-segregated Pt–Ni nanowire precursor [34]. The derived sulfide/metallic heterostructure delivered a current density of 37.2 mA cm^−2^ at the overpotential of 70 mV at pH 14, which is over 9.7 times higher than that of the commercial Pt/C. Density functional theory (DFT) calculations were employed to construct the energy diagram of the reaction. The energy barrier for breaking the H–OH bond in water was reduced from 0.89 eV on the Pt (111) surface to 0.32 eV on the NiS (100) surface, whereas the binding energy of hydrogen on Pt_3_Ni_2_ was much smaller and closer to the optimal value than that of NiS. The reaction steps thus preferentially occurred on the sites requiring less energy, leading to much increased HER activity in alkaline solution.

**Fig. 2:**
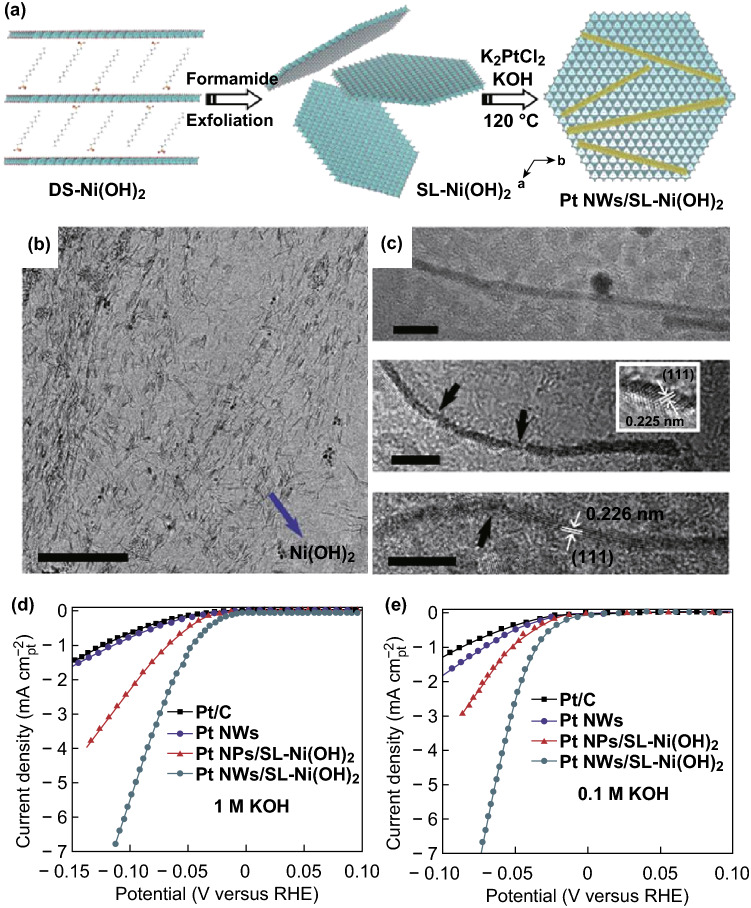
**a** Schematic of synthesis of Pt NWs/SL–Ni(OH)_2_. **b**, **c** TEM images of Pt NWs/SL–Ni(OH)_2_; scale bars in **b** and **c** are 100 and 5 nm, respectively. HER activity of Pt NWs/SL–Ni(OH)_2_, Pt NPs/SL–Ni(OH)_2_, pure Pt NWs, and commercial Pt/C (20 wt% Pt) in 1 M **d** and 0.1 M KOH **e** at room temperature. Reproduced with permission of the authors of Ref. [35]. Copyright 2015, Nature Publishing Group

Further studies focused on lowering the Pt content in the heterostructures. Tang et al. reported a hybrid nanomaterial comprising one-dimensional ultrathin Pt nanowires and single-layered nickel hydroxide (Pt nanowires/SL-Ni(OH)_2_) [35]. Control experiments revealed that the unique surface chemistry of the exfoliated single-layered Ni(OH)_2_ nanosheets profoundly affected the growth of the ultrathin platinum nanowires, with uniform diameters of about 1.8 nm and a total Pt content of 38.0 wt% (Fig. 2). This heterostructure delivered a HER current density (normalized relative to the electrochemically active surface areas (ECSAs) of the Pt species) of 2.48 mA cm^−2^ at the overpotential of 70 mV in 1 M KOH, which is almost one order of magnitude higher than that of the commercial Pt/C catalyst. Even for the mass-normalized (using Pt species) current density, the HER activity was 4.35 times that of commercial Pt/C. Similarly, Pt nanocrystals with diameters of ~ 3 nm were loaded on single-layer Ni(OH)_2_ nanosheets with a Pt content of 43 wt%, as reported by Jin and co-workers [18]. This nanocomposite, denoted as Pt@2D-Ni(OH)_2_, exhibited a fivefold improvement in the catalytic activity in 0.1 M KOH and a reduction of up to 130 mV in the overpotential compared to commercial Pt/C. It is proposed that the Pt@2D–Ni(OH)_2_ nanocomposite possesses interfaces with Pt–Ni–OH species under the HER conditions. Such interfaces facilitate both water dissociation and hydrogen recombination and account for the enhanced catalytic activity for water reduction. This group also constructed a Ni(OH)_2_–Pt/C catalyst, namely the commercial Pt/C catalyst modified with single-layer Ni(OH)_2_ nanosheets [36]. Significantly improved alkaline HER activity was recorded with the deposition of only 20 wt% single-layer Ni(OH)_2_ onto the Pt/C catalyst. Notably, atomically dispersed noble metal catalysts, also known as single-atom catalysts (SACs), exhibit competitive HER activity with extremely low noble metal contents [37, 38]. Nevertheless, to the best of our knowledge, the synthesis of SACs for the alkaline HER remains rare to date, allowing for more possibility in this promising field.

Nevertheless, due to the formation of Schottky barriers between the heterostructural interfaces, the Pt/hydroxide heterostructures often suffer from relatively low electron transfer ability. Thus, a relatively high overpotential is needed to drive the reaction. In view of this, Zheng and Wang’s group presented a Pt-decorated Ni_3_N nanosheet electrocatalyst for the HER under alkaline conditions [39]. Apart from the synergistic effect between the two components, this hybrid nanostructure also enables high electron conductivity without Schottky barrier formation, which is due to the metallic nature of Ni_3_N. As a result, the Ni_3_N/Pt composite with a Pt content of ~ 15 wt% exhibited performance ranking among the best for the HER under alkaline conditions, with a current density of 200 mA cm^−2^ at an overpotential of 160 mV, a Tafel slope of 36.5 mV dec^−1^, and excellent stability with 82.5% current retention after 24 h of operation. This work provided an effective strategy for further enhancing the catalytic performance of these heterostructured catalysts.

**Fig. 3:**
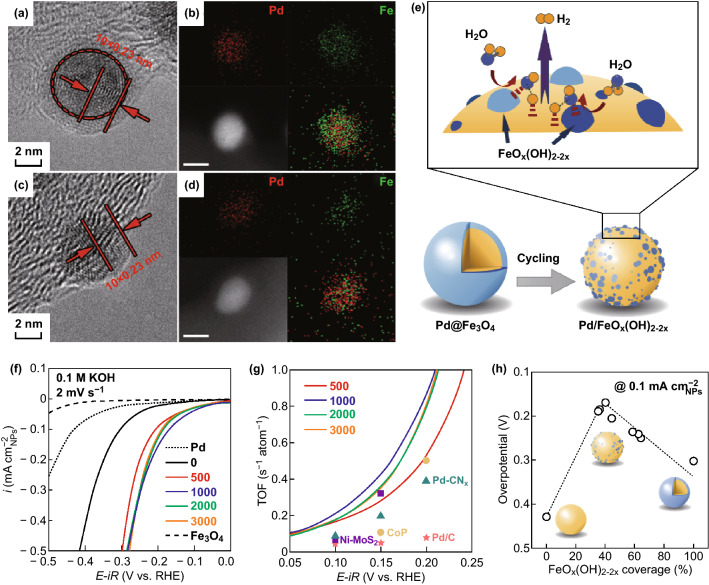
TEM images and EDS elemental mapping of Pd/FeO_*x*_(OH)_2−2*x*_ NPs **a**, **b** before cycling and **c**, **d** after 1000 electrochemical cycles. The scale bar is 5 nm. **e** Schematic diagram of the alkaline HER reaction process on the surface of core/shell Pd/FeO_*x*_(OH)_2−2*x*_ nanoparticles after electrochemical cycling. **f** *iR*-corrected linear sweep voltammogram (LSV) curves. **g** Turnover frequencies (TOFs) of Pd/FeO_*x*_(OH)_2−2*x*_ NPs and catalysts in the benchmark literature: Pd/C, Pd-CN_*x*_, Ni-MoS_2_, and CoP. **h** HER activity versus FeO_*x*_(OH)_2−2*x*_ coverage [40]. Copyright 2017, Wiley–VCH

Apart from Pt, other precious metals such as palladium and ruthenium have also been employed in heterostructured catalysts [40, 41]. Xu et al. fabricated a core-shell-structured Pd/FeO_*x*_(OH)_2−2*x*_ composite by electrochemical cycling of the starting Pd/Fe_3_O_4_ nanoparticles (Fig. 3a–d) [40]. In this hybrid, the Fe species act as additional sites for water dissociation for improving the proton supply, and the Pd surface serves as the hydrogen adsorption/desorption sites (Fig. 3e). Furthermore, they revealed that the HER activity of Pd/FeO_*x*_(OH)_2−2*x*_ depends on the coverage of iron (oxy)hydroxide on the Pd surface, which can be subtly tuned by varying the number of cycles in the electrochemical cycling process. The plot of the intrinsic alkaline HER activity as a function of the FeO_*x*_(OH)_2−2*x*_ coverage was characterized by a striking volcano shape (Fig. 3h). The optimized Pd/FeO_*x*_(OH)_2−2*x*_ composite with a FeO_*x*_(OH)_2−2*x*_ coverage of about 40% displayed a HER activity that was nineteen times greater than that of the plain Pd nanoparticles (Fig. 3f). In addition, the HER turnover frequency (TOF) of the Pd/FeO_*x*_(OH)_2−2*x*_ composite was 0.50 s^−1^ atom^−1^ at an overpotential of 150 mV, which is twenty-five times higher than that of pure Pd (0.02 s^−1^ atom^−1^) (Fig. 3g). This strategy is expected to be extendable to optimizing the activity of other multistep catalysis processes on nanosized surfaces.

### Non-Noble-Metal-Based Heterostructured Catalysts

The scarcity and high cost of noble metals restrict their large-scale utilization in catalysis. Therefore, a number of emerging studies have reported the design and development of noble-metal-free catalysts for use under alkaline conditions. In 2014, Dai’s group fabricated a NiO/Ni nanohybrid anchored on carbon nanotubes having numerous exposed NiO/Ni nanointerfaces [42]. A high HER catalytic activity close to that of commercial Pt/C catalysts was achieved in basic solution, exceeding that of the control samples without oxide/metal interfaces. The authors further illustrated the significance of the NiO/Ni interface in alkaline HER: The OH^−^ generated by H_2_O splitting preferentially attached to the positively charged Ni species in NiO, while the nearby metallic Ni sites facilitated H adsorption to generate H_2_ on the electrode. The superb HER catalytic activity was therefore attributed to the synergistic effect between the nickel species with different valences. Additionally, the presence of carbon nanotubes afforded the catalyst high conductivity for electron transfer, and rich active sites, by impeding aggregation of the nickel species.

**Fig. 4:**
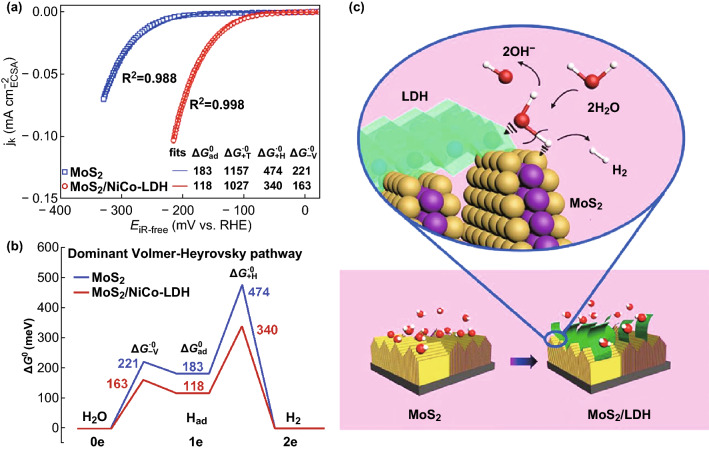
**a** ECSA-normalized polarization curves (symbols) of bare MoS_2_ and MoS_2_/NiCo-LDH composite catalysts in 1 M KOH solution with the best fits (lines) using the dual-pathway kinetic model. The fitted standard activation free energies are presented in units of meV. **b** Free energy diagram of the dominant Volmer–Heyrovsky pathway for HER in alkaline electrolyte for bare MoS_2_ (blue) and MoS_2_/NiCo-LDH composite (red) catalysts. **c** Schematic illustration of the HER at MoS_2_/LDH interface in alkaline environment. Synergistic chemisorption of H (on MoS_2_) and OH (on LDH) for enhancing the water dissociation step. Reproduced with permission of the authors of Ref. [44]. Copyright 2017, Cell Press. (Color figure online)

In the past decade, molybdenum disulfide (MoS_2_) has been recognized as a substitute for Pt for acidic HER catalysis due to its adequate ability to adsorb hydrogen species on its edge sites [43]. For the alkaline HER, the catalytic performance of MoS_2_-based heterostructured catalysts can also be promoted by accelerating the Volmer step. For instance, Ni(OH)_2_ nanoparticles were electrodeposited on the surface of MoS_2_ nanosheets that were previously vertically grown on conductive carbon cloth [19]. This hybrid nanostructure exhibited an overpotential of 80 mV at 10 mA cm^−2^ and a Tafel slope of 60 mV dec^−1^ in 1.0 M KOH electrolyte, both of which surpassed those of the unitary counterparts. The authors claimed that the increased activity of the hybrid catalyst was ascribed not only to the interfacial cooperation between Ni(OH)_2_ and MoS_2_, but also to charge transfer from Ni(OH)_2_ to MoS_2_, leading to a more optimal binding energy for the reaction intermediates. In another study, Yang et al. assembled a MoS_2_/NiCo-layered double hydroxide (LDH) heterostructure as a HER electrocatalyst for use in alkaline electrolyte, where the heterostructure exhibited an extremely low overpotential of 78 mV at 10 mA cm^−2^ and a low Tafel slope of 76.6 mV dec^−1^ in 1 M KOH solution [44]. For both MoS_2_ and MoS_2_/NiCo-LDH, the reaction proceeds through a Volmer–Heyrovsky pathway. The activation energy of the Heyrovsky and Volmer steps in the reactions over the MoS_2_/NiCo-LDH catalyst was much lower than that with bare MoS_2_, as hybridization accelerates the water dissociation steps in the HER in alkaline environment (Fig. 4). Long-term stability is another key parameter for evaluating the performance of an electrocatalyst. For instance, Liu’s group synthesized a heterostructure, where Co(OH)_2_ nanoparticles were confined in MoS_2_ nanosheets [45]. The catalyst displayed a low onset overpotential of 15 mV, a small Tafel slope of 53 mV dec^−1^, and good durability (sustained for 20-h test). Here, the unique sandwich-like structure was considered to prevent the aggregation or loss of Co(OH)_2_, therefore leading to good stability.

Apart from the intrinsic catalytic activity, the electron transfer ability and the density of active sites in the electrode also impact the catalytic performance. For heterostructured catalysts, such as MoS_2_ nanosheet-supported Ni/Co hydroxides, the catalytic performance is limited by two features: (1) The poor electronic conductivity of 2H-MoS_2_ fails to afford fast electron transfer and (2) the number of catalytic sites on 2H-MoS_2_ is insufficient as only the edge sites are active for the HER. In light of these considerations, Liang et al. fabricated a Ni oxyhydroxide/1T-MoS_2_ hybrid by in situ growth of Ni oxyhydroxide nanoparticles on 1T-MoS_2_ nanosheets [46]. The optimized catalyst was obtained by tuning the content of the Ni species: A cathodic current density of 10 mA cm^−2^ at an overpotential of 73 mV, which is 185 mV less than that of the original 1T-MoS_2_ in 1 M KOH, was achieved. The remarkable activity of this nanohybrid originated from multiple features of the structure: the synergistic effect between Ni oxyhydroxide and 1T-MoS_2_, the metallic nature of 1T-MoS_2_ that facilitates electron transfer, and the active basal planes of 1T-MoS_2_ that dramatically increase the number of catalytic sites.

Nevertheless, 1T-MoS_2_ is neither readily synthesized, nor stable under harsh catalytic conditions due to its metastable nature. Alternatively, 2H-MoS_2_ attached to conductive nickel/cobalt sulfides has recently been fabricated. Feng et al. presented a nickel form-supported MoS_2_/Ni_3_S_2_ heterostructure, in which the outer MoS_2_ nanosheets were decorated on the surface of the inner Ni_3_S_2_ nanoparticles. In 1 M KOH, the overpotential required to achieve a current density of 10 mA cm^−2^ was approximately 110 mV [47]. Interestingly, this heterostructure also exhibited a low overpotential for the oxygen evolution reaction in the same electrolyte. Employing this MoS_2_/Ni_3_S_2_ heterostructure as a bifunctional electrocatalyst, the resulting alkali electrolyzer delivered a current density of 10 mA cm^−2^ at the very low cell voltage of 1.56 V. An overpotential of 204 mV at current density of 10 mA cm^−2^ in alkaline solution was also obtained with a NiS_2_/MoS_2_ hybrid synthesized by chemical vapor sulfurization of NiMoO_4_ nanowires, as reported by Yu and co-workers [48].

## Emerging Strategies for Accelerating the Volmer Step

Thus far, heterostructured catalysts have shown superior performance in alkaline HER catalysis, as demonstrated by a large number of examples. Theoretically, improvements in both the Volmer step and Heyrovsky/Tafel step should enhance the catalytic efficiency of the overall process. Nevertheless, for the heterostructured catalysts reported so far, the Volmer step was revealed to be the rate-determining step on most of these interfaces [36, 49, 50]. Therefore, accelerating the Volmer step is considered to be a path for directly increasing the catalytic performance [51]. In 2012, after discovering the synergistic effect of the Pt/Ni(OH)_2_ heterostructure, Markovic and co-workers investigated the relation between the catalytic activity and the Volmer step for several 3d-M hydroxides (3d-M = Mn, Fe, Co, and Ni) by constructing a series of Pt/3d-M hydroxide hybrids [25]. It was found that the catalytic ability of these heterostructures depends on their affinity for OH^−^, where strong affinity leads to the accumulation of adsorbed OH^−^ on the surface, thus inhibiting the adsorption of another H_2_O in the following reaction. Based on the experimental results, they proposed the following affinity trend: Ni < Co < Fe < Mn, which is converse to the catalytic activity of the corresponding Pt/3d-M hydroxide hybrids. Nickel hydroxides show the weakest affinity for OH^−^; thus, the corresponding heterostructure exhibited the best catalytic activity in alkaline solution. This trend was further confirmed for the 1T-MoS_2_/3d-M hydr(oxy)oxide (3d-M = Fe, Co, and Ni) heterostructures, as reported by Liang and co-workers [46]. However, Zhao’s group has recently reported a discrepant activity trend. In their research, the monolayer MoS_2_ nanosheets hybridized with Co(OH)_2_ exhibited enhanced HER activity compared to that using Ni(OH)_2_ under identical synthesis and test conditions [52]. Therefore, more effort should be devoted to this point to establish the intrinsic trends in the catalytic ability based on the Volmer step of these 3d-metal hydroxides.

**Fig. 5:**
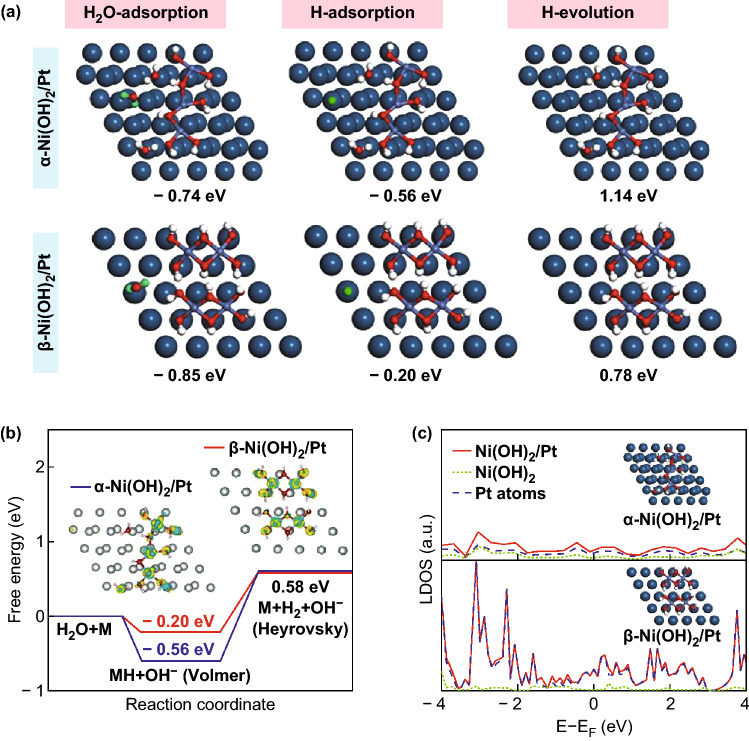
**a** Free energies of H_2_O adsorption, H adsorption, and H_2_ evolution on *α*- or *β*-Ni(OH)_2_/Pt electrode; for clarity, the adsorbed H atoms for the H_2_O molecules in the top site on the Pt surfaces are colored green. **b** Adsorption free energy diagram for the Volmer and Heyrovsky steps. **c** LDOS of the *α*- or *β*-Ni(OH)_2_/Pt electrode. Reproduced with permission of the authors of Ref. [53]. Copyright 2018, American Chemical Society. (Color figure online)

Because nickel hydroxides display favorable water dissociation ability, the role of the Volmer step in the catalytic activity of nickel hydroxides with different polymorphs was investigated by Shi and Ma’s group [53]. They prepared *α*- or *β*-Ni(OH)_2_/Pt hybrids by loading different nickel hydroxides on the surface of a Pt electrode and found that the *β*-Ni(OH)_2_/Pt electrode exhibited superior activity and reaction kinetics for the HER in alkaline media compared to the *α*-Ni(OH)_2_/Pt electrodes (Fig. 5). On the basis of experimental results and DFT calculations, the superb HER activity of the *β*-Ni(OH)_2_/Pt electrode was ascribed to the structural features of *β*-Ni(OH)_2_, which possesses a larger interlamellar spacing than *α*-Ni(OH)_2_, resulting in more facile access to the sites for adsorbing water molecules and desorbing OH^−^ ions. Interestingly, it was revealed that the interaction between *β*-Ni(OH)_2_ and Pt was stronger than that of α-Ni(OH)_2_. Such an electronic effect provides active sites on the Pt surface with a more suitable binding energy for the H intermediates. Similar electronic effects were observed between the two components in the MoS_2_/Ni(OH)_2_ and MoS_2_/NiCo-LDH heterostructures [19, 44]. Charge transfer from Ni(OH)_2_ to MoS_2_ on the Ni(OH)_2_/MoS_2_ interface was confirmed by DFT calculations and led to a more optimal Δ*G*_H_ value of about − 0.06 eV for the MoS_2_ edge sites, thereby accelerating subsequent H_2_ generation.

**Fig. 6:**
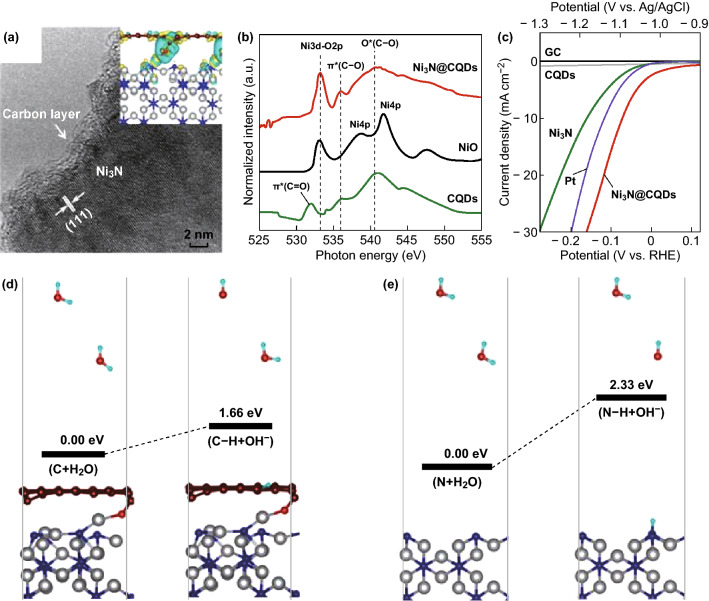
**a** High-resolution TEM (HRTEM) image of Ni_3_N@CQDs, **b** O K-edge X-ray absorption near-edge structure (XANES) spectra of Ni_3_N@CQDs, commercial NiO and the CQDs treated in NH_3_ at 370 °C, **c** linear scan voltammetry (LSV) polarization curves of Ni_3_N@CQDs in comparison with those of platinum (Pt) electrode, pristine Ni_3_N, CQDs, and glassy carbon (GC) electrode in a 1 M KOH aqueous solution. Comparison of HER Volmer reaction step and the resultant binding energies on carbon-coated Ni_3_N(110) **d** and pristine Ni_3_N(110) **e** surfaces. N, Ni, C, O, and H atoms are indicated by blue, gray, brown, red, and cyan, respectively. Reproduced with permission of the authors of Ref. [54]. Copyright 2018, American Chemical Society. (Color figure online)

Lately, certain surface modification strategies, such as coating, have been developed to provide satisfactory surfaces for the Volmer step in the alkaline HER. Zhou et al. [54] sought to circumvent the water dissociation step by constructing a polarized carbon surface on Ni_3_N nanoparticles (Ni_3_N@CQDs). The researchers created carbon-reinforced Ni_3_N by dipping Ni(OH)_2_ in a carbon quantum dot solution and then heating the recovered solids to convert the Ni(OH)_2_ to Ni_3_N (Fig. 6). The activation energy of the Volmer step was significantly lowered on the charge-polarized carbon surface, resulting in enhanced catalytic activity, with an overpotential of 69 mV at a current density of 10 mA cm^−2^ in a 1 M KOH aqueous solution, which is significantly lower than that of the Pt electrode under the same conditions. The carbon coating was also found to protect the interior of the Ni_3_N layer from oxidation/hydroxylation over hours of continuous use.

Recent studies have also suggested that decoration with copper can alter the electronic structure of 3d-metal-based materials to facilitate faster kinetics in the Volmer step. Zou’s group reported a copper cluster-coupled cobalt sulfide heterostructure [55]. Strong charge redistribution was found to occur at the interface region. Electron transfer from Co to Cu led to increased electron density in the Cu cluster region, along with depletion of the electrons at the interfacial cobalt atom region. The formed positive and negative regions in turn strongly promoted the water dissociation step, further facilitating the surface water splitting reaction kinetics. Similarly, Li and co-workers synthesized Cu nanodot-decorated Ni_3_S_2_ nanotubes as efficient electrocatalysts for the HER in alkaline media [56]. The electronic interaction between Cu and Ni_3_S_2_ led to positively charged Cu and negatively charged Ni_3_S_2_. The former can effectively adsorb and activate water molecules and promote H–O cleavage, while the latter can weaken the S–H_ads_ bonds and thus should enhance H adsorption and desorption. As a result, this Cu/Ni_3_S_2_ hybrid exhibited obviously improved electrocatalytic activity and durability for the HER, such as a low onset overpotential of 60 mV, a low overpotential of 128 mV at 10 mA cm^−2^, and excellent durability with a current density increase of only ~ 3% at an overpotential of 250 mV over 30 h. These studies provide new strategies for optimizing the energy of adsorption/desorption of the HER intermediates on the surface of catalysts in alkaline media.

## Summaries and Perspectives

Recent trends have highlighted the rational design of heterostructured materials for obtaining highly efficient HER electrocatalysts with intriguing synergistic effects in alkaline solutions. For Pt-based heterostructured catalysts, studies have been conducted by either enlarging the interfaces, or lowering the content of Pt species in the heterostructures, resulting in specific activities superior to that of the benchmark Pt/C catalyst. For noble-metal-free catalysts, Ni/Co hydroxides and MoS_2_ are the commonly utilized building blocks in the heterostructures, leading to enhanced performance through optimization of the density of the catalytic sites and the electron/mass transfer efficiency of the electrode. In addition, acceleration of the Volmer step via various strategies has been demonstrated to be a straightforward route for achieving improved activity of catalysts for the alkaline HER. Table 1 summarizes recently developed heterostructured catalysts for the HER in alkaline solutions. Note that the recorded activities are affected by many factors, including the noble metal content of the catalysts, the exposed surface area, and the utilization or lack of 3D conductive substrates such as nickel foam and carbon fiber cloth within the electrode [57, 58]. It should also be noted that it is impossible to compare all of these catalysts in terms of the intrinsic activities due to the lack of information on the surface area and catalyst loading.

**Table 1:** Performance of reported heterostructured catalysts for HER in alkaline medium

Catalysts	Electrolyte	Substrate	Overpotential (current density by electrode surface area)	Overpotential (current density by catalyst surface area)	Pt content	Tafel slope (mV dec^−1^)	Refs.
*Platinum*-*based heterostructures*							
NiO_*x*_/Pt–Ni	1 M KOH	GCE^a^	70 mV@19.8 mA cm^−2^	N/A	15.3 μg cm^−2^	N/A	[33]
Pt–Ni/NiS	1 M KOH	GCE	70 mV@37.2 mA cm^−2^	N/A	15.3 μg cm^−2^	N/A	[34]
Pt nanowires/SL-Ni(OH)_2_	1 M KOH	GCE	70 mV@10.9 mA cm^−2^	70 mV@2.48 mA cm^−2^	38 wt%	N/A	[35]
	0.1 M KOH		70 mV@25.2 mA cm^−2^	70 mV@6.31 mA cm^−2^			
Pt nanocrystals @2D-Ni(OH)_2_	0.1 M KOH	GCE	100 mV@5 mA cm^−2^	N/A	43 wt%	72	[18]
Ni(OH)_2_–Pt/C	0.1 M KOH	GCE	157 mV@5 mA cm^−2^	N/A	20 wt%	N/A	[36]
Ni_3_N/Pt	1 M KOH	Ni Foam	160 mV@200 mA cm^−2^	N/A	15 wt%	36.5	[39]
Pt–Co(OH)_2_	1 M KOH	Carbon Cloth	32 mV@10 mA cm^−2^	200 mV@3 mA cm^−2^	5.7 wt%	70	[61]
PtO_2_–Co(OH)F	0.1 M KOH	Ti mesh	39 mV@4 mA cm^−2^	100 mV@145 μA cm^−2^	4.8 wt%	63	[62]
PtO_2_–CoOOH	1 M KOH	Ti mesh	14 mV@10 mA cm^−2^	N/A	N/A	39	[63]
Pd/FeO_*x*_(OH)_2−2*x*_	0.1 M KOH	GCE	280 mV@5 mA cm^−2^	150 mV@0.1 mA cm^−2^	N/A	131–162	[40]

Catalysts	Electrolyte	Substrate	Overpotential (current density by electrode surface area)	Overpotential (current density by catalyst surface area)	Tafel slope (mV dec^−1^)	Refs.
*Non*-*noble-metal*-*based heterostructures*						
NiO/Ni-CNT	1 M KOH	GCE	< 100 mV@10 mA cm^−2^	100 mV@0.02 mA cm^−2^	82	[42]
Ni(OH)_2_/MoS_2_	1 M KOH	Carbon cloth	80 mV@10 mA cm^−2^	N/A	60	[19]
MoS_2_/NiCo-LDH	1 M KOH	CFP^b^	78 mV@10 mA cm^−2^	200 mV@0.1 mA cm^−2^	76.6	[44]
MoS_2_-confined Co(OH)_2_	1 M KOH	GCE	89 mV@10 mA cm^−2^	N/A	53	[45]
1T-MoS_2_/NiOOH	1 M KOH	GCE	73 mV@10 mA cm^−2^	N/A	75	[46]
MoS_2_/Ni_3_S_2_	1 M KOH	Ni Foam	110 mV@10 mA cm^−2^	N/A	83	[47]
NiS_2_/MoS_2_	1 M KOH	GCE	204 mV@10 mA cm^−2^	204 mV@0.1 mA cm^−2^	65	[48]
MoSe_2_@Ni_0.85_Se	1 M KOH	GCE	117 mV@10 mA cm^−2^	117 mV@2.1 μA cm^−2^	66	[64]
2D-MoS_2_/Co(OH)_2_	1 M KOH	GCE	128 mV@10 mA cm^−2^	128 mV@0.4 mA cm^−2^	76	[52]
Ni_3_N@CQDs	1 M KOH	GCE	69 mV@10 mA cm^−2^	69 mV@0.48 mA cm^−2^	108	[54]
Cu@CoS*x*	1 M KOH	CFP	134 mV@10 mA cm^−2^	N/A	N/A	[55]
Cu NDs/Ni_3_S_2_	1 M KOH	CFP	128 mV@10 mA cm^−2^	128 mV@6.4 μA cm^−2^	76.2	[56]

^a^*GCE* Glassy carbon electrode

^b^*CFP* Carbon fiber paper

Although remarkable progress has been achieved in the field of alkaline HER catalysis due to the emergence of heterostructured materials, there is still room for the development of better catalysts. To achieve lower cost, higher stability, and higher efficiency, next-generation catalysts must overcome several limitations. First, although the specific catalytic activity of most Pt-based heterostructures surpasses that of the Pt/C catalysts, no significant reduction in the Pt content in these materials has been realized to date. The synthesis of single-atom catalysts (SACs) with suitable substrates on the nanoscale or microscale has recently emerged and may provide an avenue for lowering the Pt loading [37, 59]. Thus, the electronic configuration and catalytic activity of Pt-based SACs within different chemical environments have to be identified. Second, in spite of the impressive progress in the development of noble-metal-free heterostructures, thus far, the catalytic performance of these species does not satisfy industrial requirements. In general, catalysts with numerous active sites and favorable ability for electron and mass transfer should offer better catalytic activity [60]. Specifically, for 2D nanocatalysts such as MoS_2_ nanosheets where the active sites are confined within the edge atoms, precise control of the surface and interface is needed to tune the catalytic performance of these nanosized heterostructures. This represents a material engineering challenge. Third, for most of the heterostructured catalysts, the Volmer step is the rate-determining step in the alkaline HER. However, few strategies focusing on this point have been reported so far. Studies combining experimental results and theoretical calculations should be conducted with the aim of fabricating heterostructured catalysts with acceleration of the Volmer step in the alkaline HER, which may provide more opportunities in the materials research community.
